# Prevalence, serotype, and antimicrobial resistance profiles of children infected with *Salmonella* in Guangzhou, southern China, 2016–2021

**DOI:** 10.3389/fped.2023.1077158

**Published:** 2023-03-15

**Authors:** Fei Gao, Zhenting Huang, Zhile Xiong, Hao Zheng, Qiulian Deng, Huamin Zhong, Sufei Zhu, Yan Long, Jielin Wang

**Affiliations:** Clinical Laboratory Department, Guangzhou Women and Children's Medical Center, Guangzhou Medical University, Guangdong Provincial Clinical Research Center for Child Health, Guangzhou, China

**Keywords:** children, salmonella, bacterial resistance rate, multi-drug resistant, serotypes

## Abstract

**Purpose:**

*Salmonella* infection is a key global public health concern and has lead to an increased economic burden on society. We investigated the epidemiological characteristics and antimicrobial resistance profiles of clinically isolated *Salmonella* strains in Guangzhou Women and Children's Medical Center.

**Patients and methods:**

This was a retrospective study of 1,338 *Salmonella* strains collected from children in Guangzhou Women and Children's Medical Center during 2016 to 2021.

**Results:**

The results revealed that 1,338 cases of *Salmonella* were mainly isolated from feces and blood samples. The age distribution was dominated by infants under 3 years old. The seasonal distribution was high in summer and autumn. 48 serotypes were detected, and *S. typhimurium* (78.7%) was the predominant serogroup. The results of antimicrobial susceptibility showed that the highest resistance was observed in ampicillin (84.5%), while lower resistance was observed in piperacillin/tazobactam, cefoperazone/sulbactam and ciprofloxacin. The antimicrobial resistance rate of fecal isolates was higher than that of blood isolates. The five-year average detection rate of multi-drug resistant *Salmonella* was 8.5% (114/1338) and the MDR rate of *S. typhimurium* was the lowest (6.9%; 73/1053).

**Conclusion:**

We concluded that antibacterial treatment should be carefully selected according to serotype and antimicrobial sensitivity results in children. Antimicrobial resistance monitoring for multi-drug resistant *Salmonella* is still required.

## Introduction

*Salmonella* is a rod-shaped, gram-negative bacterium with no capsule and spore and is a member of the *Enterobacterales* (1). It can be transmitted *via* contaminated food products including meat, beef, potatoes and cucumbers, which may lead to foodborne outbreaks, and results in about 23,000 deaths every year (2–4). Moreover, *Salmonella* is the main pathogenic cause of diarrhea in children, with the largest proportion of infections in pediatric patients < 5 years old and most commonly occurs between summer and autumn (5, 6). Currently, more than 2,500 *Salmonella* serotypes have been reported and among them, over 20 serotypes can cause zoonotic diseases (7). *S. typhimurium* and *S. enteritidis* are the major *Salmonella* serotypes responsible for gastroenteritis and diarrhea in both children and adults (8).

The pathogenicity of *Salmonella* is mainly related to its toxic factors, including pathogenic islands, toxic plasmids, pili and enterotoxins (9, 10). Different serotypes carrying different virulence genes can exhibit different sensitivity to antibiotics (11). In recent years, studies have shown that *Salmonella* antibiotic resistance has markedly increased, due to long-term antibiotic use in the poultry industry and animal laboratory products (12, 13). Furthermore, there are many contraindications to clinical medication for children, which brings great challenges to clinical diagnosis and treatment. In order to understand *Salmonella* serotype distribution and antimicrobial resistance in Guangzhou, Southern China and to identify potential control strategies for formulating prevention and treatment plans, a retrospective study of 1,338 *Salmonella* strains isolated in Guangzhou Women and Children's Medical Center (National Children's Medical Center for South Central Region) from 2016 to 2021 was conducted in this study.

## Materials and methods

### Ethical approval

This study has been approved by the Ethics Committee of Guangzhou Women's and Children's Medical Center (No. 176A01, 2021).

### Sample, clinical data collection and isolation of *Salmonella* strains

Between January 2016 and December 2021, 1,388 *Salmonella* were isolated from 12,361 pediatric patients samples at Guangzhou Women and Children's Medical Center (National Children's Medical Center for South Central Region). Clinical information, including gender, age, main clinical manifestations, laboratory examinations, etiology, and antimicrobial sensitivity test results was collected in electronic medical review system from January 2016 to December 2021.

The diagnostic criteria for diarrhea refer to the “Standards for the Diagnosis and Treatment of Acute Infectious Diarrheal Diseases in Children” issued in 2020. Within 2 weeks of the disease course, changes in fecal characteristics and increased fecal frequency can be diagnosed for acute diarrhea. Exclusion criteria is 1) incomplete clinical medical records; 2) children with diarrhea caused by nosocomial infection during hospitalization due to other diseases; 3) if the same child has the same strains isolated from the multiple fecal culture during the same hospitalization period, only the first result was included for analysis, and duplicate strains was eliminated.

### *Salmonella* identification and antimicrobial susceptibility testing

*Salmonella* culture was performed according to the methods previously described (14). Briefly, clinical samples were inoculated in culture plates. Then, suspected colonies were tested using standard biochemical methods and colonies considered to be *Salmonella* had the following biochemical phenotypes: growth on tripler-sugar-iron agar (acid from glucose, gas, production of H2S); negative for lactose, sucrose, urease, oxidase,salicin, -galactosidase and indole production; positive for nitrate reduction and lysine decarboxylase (14). Matrix-Assisted Laser Desorption Ionization-Time of Flight Mass Spectrometry (MALDI-TOF-MS) and VITEK 2-Compact automatic bacterial identification antimicrobial susceptibility system were used for identification and antimicrobial susceptibility testing(ampicillin; piperacillin/ tazobactam; aztreonam; ceftazidime; ceftriaxone; cefotaxime; cefepime; cefoperazone/sulbactam; ciprofloxacin; imipenem; chloramphenicol). The K-B antimicrobial susceptibility test method and Clinical and Laboratory Standards Institute (CLSI, 2021) on Antimicrobial Susceptibility Testing breakpoints were used to assess the results. *Escherichia coli* ATCC 25,922, *Pseudomonas aeruginosa* ATCC 27,853 and *Salmonella* ATCC 14,028 (all from the Clinical Laboratory Center of the Ministry of Health) were used as the quality control strains for validation of antimicrobial susceptibility testing. Multidrug-resistant (MDR) strains were defined by resistance to three or more antimicrobial classes.

### Salmonella serotyping

*Salmonella* isolates serotyping was conducted by using a classic slide agglutination assay with anti-*Salmonella* (A∼F), anti-O and anti-H serum (Tianrun Bio-Pharmaceutical, Ningbo, China). We used Phosphate Buffered Saline (PBS) as negative control and *Salmonella* ATCC 14,028 as positive control.

### Statistical analysis

WHONET 5.6 was used to analyze antimicrobial susceptibility data. SPSS 23.0 was used for statistical analysis. Briefly, we performed a normality test on the measurement data, and those that did not conform to the normal distribution were described by the median (interquartile range). The counting data was expressed as number of cases and percentages. The comparison used *χ*^2^ -value test or Fisher's exact probability method. *P* < 0.05 indicates statistically significant differences.

## Results

### Clinical information and distribution of *Salmonella* isolates

**Figure 1:**
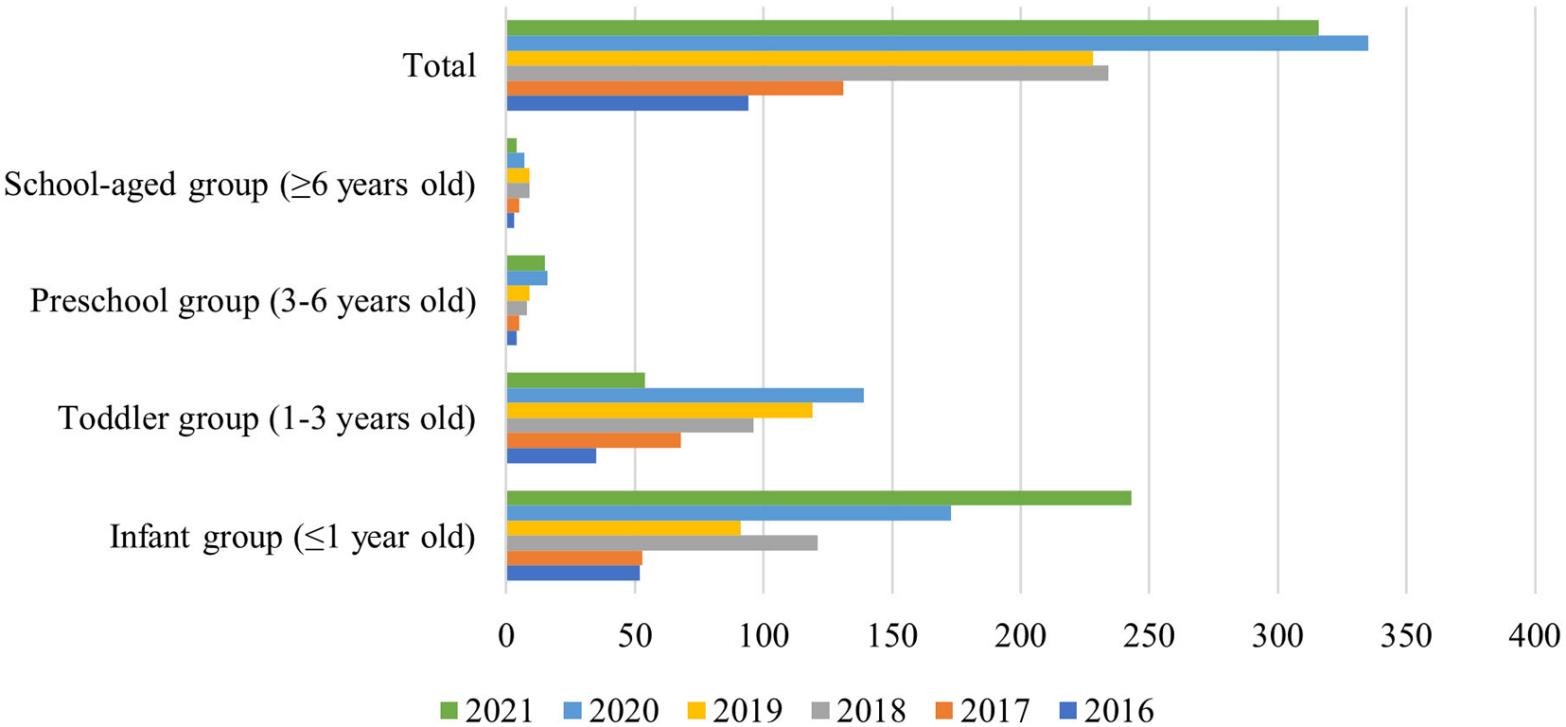
Age distribution of children with 1,338 *Salmonella* strains. Children are divided into four groups by age. The different bars represent different years. Age distribution of children with *Salmonella* infection was mostly under 3 years old.

**Figure 2:**
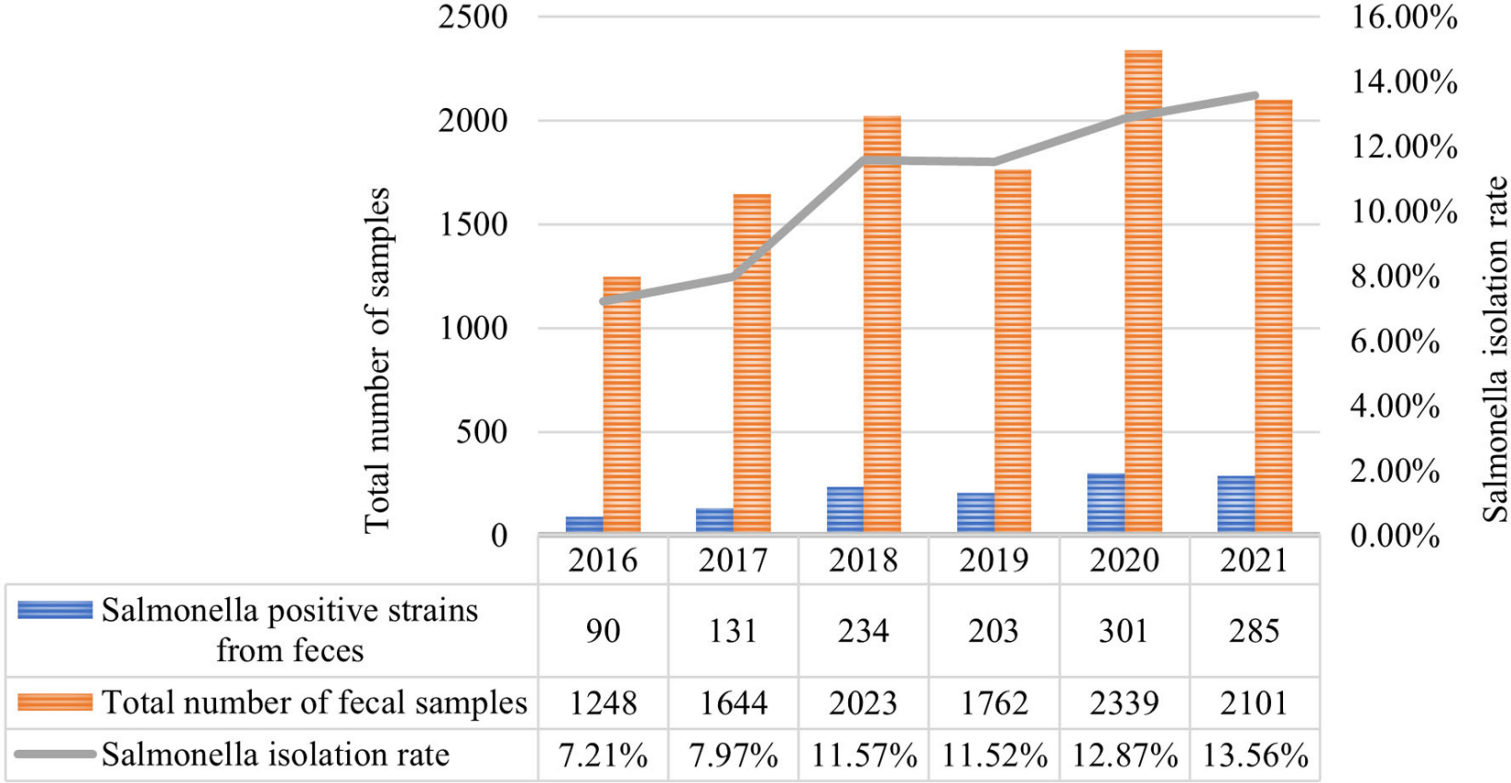
Annual distribution of *Salmonella* isolated from feces from 2016 to 2021. The bar chart represents the total number of fecal samples and *Salmonella* positive strains from feces. The *Salmonella* isolation rate of 6 years is shown in the line graph. *Salmonella* exhibited an increasing trend year by year.

From January 2016 to December 2021, a total of 1,338 *Salmonella* strains were isolated from 12,361 children samples at in Guangzhou Women and Children's Medical Center (Figure 2). As shown in Figure 1, the majority of *Salmonella* isolates were collected from children under 3 years old [92.9% (1244/1338)], with an average age of 13 (6.3–34.7) months (Table 2). The isolate numbers of all serogroups increased dramatically over time. The isolate number in 2020 was 335 strains and 316 strains in 2021 (Figure 1). As shown in Table 1, the total isolation number was 93 in spring, 356 in summer, 608 in autumn, and 281 in winter.

**Table 1:** Seasonal distribution of 1,338 Salmonella strains.

Seasons	2016	2017	2018	2019	2020	2021	Total
Spring (Jan. to Mar.)	5	19	6	22	13	28	93
Summer (Apr. to Jun.)	19	39	76	63	74	85	356
Autumn (Jul. to Sept.)	55	43	105	99	171	135	608
Winter (Oct. to Dec.)	15	30	47	44	77	68	281
Total	94	131	234	228	335	316	1338

**Table 2:** The clinical information of 1,338 Salmonella isolates.

Variable	Value
Sex	*n*^a^ (%)
Male	826 (61.73%)
Female	512 (38.27%)
Source	*n* (%)
Inpatient	670 (50.07%)
Outpatient	668 (49.93%)
Age	Median (range)^b^
	13 (6.3–34.7)
Clinical symptoms	*n* (%)
Fever	832 (62.18%)
Diarrhea	805 (60.16%)
Fever and diarrhea	363 (27.13%)
Vomiting	598 (44.69%)
Respiratory symptoms	164 (12.25%)
Leukemia	72 (5.38%)

^a^ Represents the number of strains.

^b^ Represents months.

Among the isolated strains, 92.8% (1241/1338) of strains were collected from feces, 5.2% (71/1338) from blood, 1.0% (13/1338) from pus, and 1.0% (13/1338) from other kinds of samples (Figure 2). As shown in Table 2, 670 strains were isolated from inpatients and 668 strains from outpatients; 826 from male and 512 from female (ratio 1.6:1). Symptoms were mainly fever and diarrhea, with some patients experiencing vomiting, respiratory infection and leukemia (Table 2).

### Distribution of serotypes for *Salmonella* isolates

**Figure 3:**
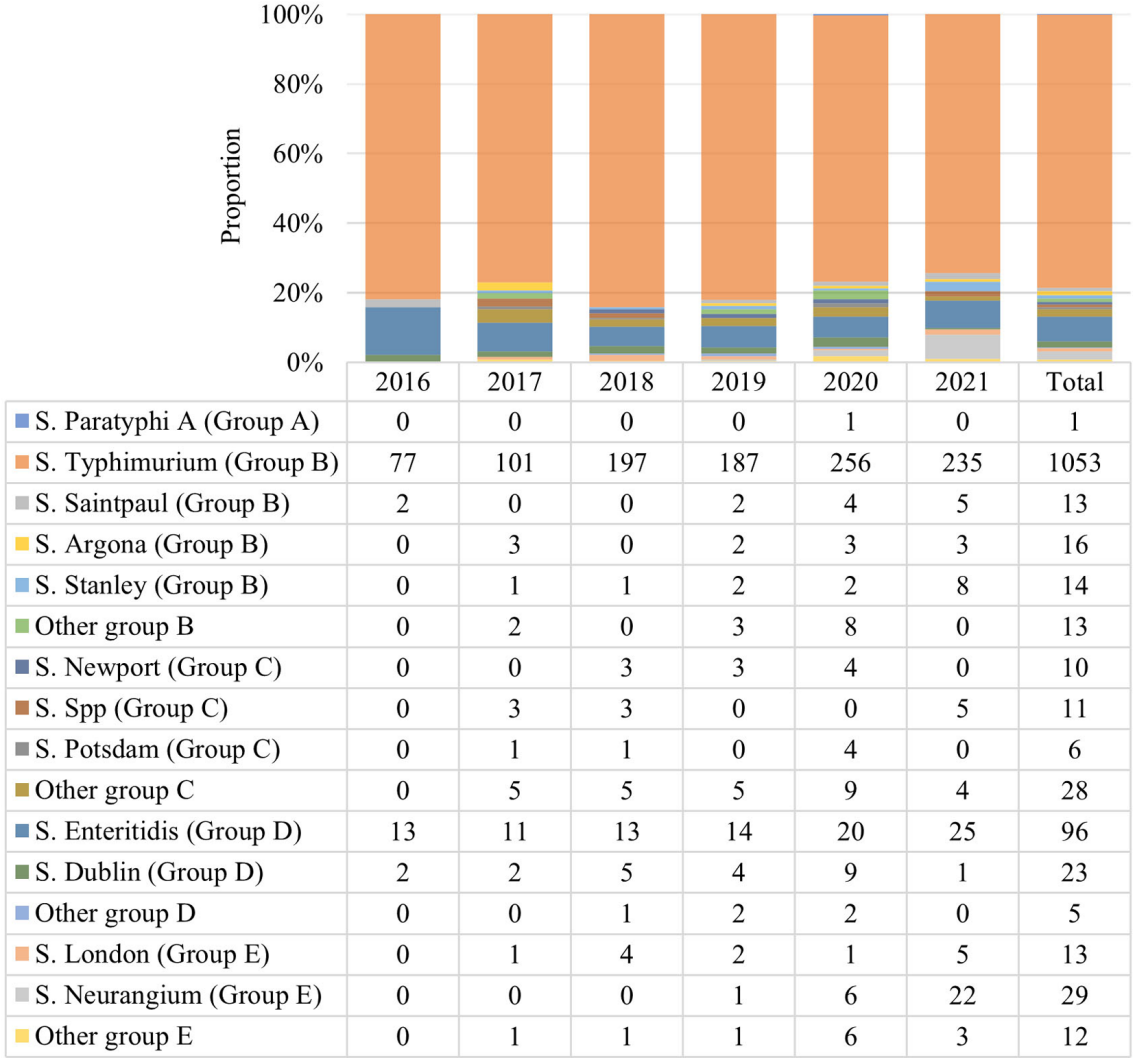
Serotype distribution of 1,338 *Salmonella* strains. 48 *Salmonella* serotypes were detected. This chart displays the major serotypes with different bar colors. Groups B and D were the top 2 predominant serogroups and *S. Typhimurium* was the predominant strain.

Among the identified 1,338 *Salmonella* isolates, 48 serotypes were detected, with group B (1110, 82.88%) and D (124, 9.27%) the top 2 predominant serogroups. The *S. typhimurium* (group B) serotype was detected in 78.69% (1053/1338), followed by 7.17% (96/1338) of *S. enteritidis* (group D) and 2.17% (29/1338) of *S. neurangium* (group E). Other rare serotypes were also detected, including 23 strains of *S. dublin*, 16 strains of *S. Argona*, 14 strains of *S. stanley*, 13 strains each of *S. saintpaul* and *S. london*, 11 strains of *S. spp*, 10 strains *S. newport*, and 6 strains *S. potsdam* (Figure 3).

### Phenotypic antimicrobial resistance

As shown in Table 3, the resistance rate of *Salmonella* isolated from children from 2016 to 2021 to ampicillin (AMP) was 84.5%. Resistance rates against 3rd generation cephalosporins were as follows: ceftriaxone (CRO, 40.1%), cefotaxime (CTX, 35.4%), ceftazidime (CAZ, 26.7%) and cefepime (FEP, 23.5%). Lower resistance rates were observed in piperacillin/tazobactam (PIP, 4.8%), cefoperazone/sulbactam (SCF, 4.1%) and ciprofloxacin (CIP, 6.7%). All strains were sensitive to imipenem (IPM) (Table 3). 97 strains of *Salmonella* isolated from blood and sterile body fluids demonstrated higher resistance to AMP (69.7%), and lower resistance to SCF (2.3%) and PIP (1.4%). 1,241 strains of *Salmonella* isolated from feces also exhibited the highest resistance to AMP (83.6%) and the lowest to SCF (3.8%) and PIP (3.6%). For all strains, the resistance rate of fecal isolates to the tested antibiotic agent was higher than that of blood and sterile body fluid isolates (Table 4).

**Table 3:** Antimicrobial resistance rates of the 1,338 Salmonella strains.

Antibiotic agent	2016 (*n* = 94)		2017 (*n* = 131)		2018 (*n* = 234)		2019 (*n* = 228)		2020 (*n* = 335)		2021 (*n* = 316)		2016–2020 (*n* = 1338)	
	No.	R (%)	No.	R (%)	No.	R (%)	No.	R (%)	No.	R (%)	No.	R (%)	No.	R (%)
AMP	72	76.6	113	86.3	201	85.9	191	83.8	301	89.9	253	80.1	1131	84.5
ATM	0	0	47	35.9	59	25.2	73	32	69	20.6	51	16.1	299	22.30%
PIP	24	25.5	11	8.4	16	6.8	10	4.4	0	0	3	0.9	64	4.80%
CAZ	32	34	37	28.2	75	32.1	53	23.2	71	21.2	48	15.2	357	26.7
CRO	41	43.6	64	48.9	107	45.7	81	35.5	100	29.9	77	24.4	536	40.1
CTX	46	48.9	67	51.1	140	59.8	83	36.4	129	38.5	65	20.6	473	35.4
FEP	32	34	45	34.4	81	34.6	55	24.1	47	14	35	11.1	314	23.5
SCF	6	6.4	11	8.4	20	8.5	14	6.1	10	3	0	0	55	4.1
CIP	7	7.4	12	9.2	23	9.8	24	10.5	49	14.6	16	5.1	90	6.7
IPM	0	0	0	0	0	0	0	0	0	0	0	0	0	0
CHL	29	30.9	57	43.5	101	43.2	118	51.8	192	57.3	157	49.7	654	48.9

AMP, ampicillin; PIP, piperacillin/tazobactam; ATM, aztreonam; CAZ, ceftazidime; CRO, ceftriaxone; CTX, cefotaxime; FEP, cefepime; SCF, cefoperazone/sulbactam; CIP, ciprofloxacin; IPM, imipenem; CHL, chloramphenicol; R, resistant; No. represents the number of resistant strains.

**Table 4:** Analysis of antimicrobial resistance rate of Salmonella isolates from blood and feces.

Antibiotic agent	Feces (*n* = 1241)		Blood and sterile body fluids (*n* = 97)		*χ* ^2^	*P* value
	No.	R (%)	No.	R (%)		
AMP	1037	83.6	68	69.7	15.322	<0.001
ATM	299	24.1	7	7.1	1.641	0.518
PIP	45	3.6	1	1.4	5.663	0.017
CAZ	257	20.7	15	15.1	5.778	0.016
CRO	397	32	20	20.3	16.188	<0.001
CTX	417	33.6	0	0	12.856	<0.001
FEP	249	20.1	16	16.7	6.599	0.01
SCF	47	3.8	2	2.3	1.717	0.307
CIP	103	8.3	16	16	0.955	0.328
IPM	0	0	0	0	/	/
CHL	612	49.3	16	16.7	21.232	<0.001

R, resistant; No. represents the number of resistant strains.

### Multidrug-resistant (MDR) *Salmonella* isolates

A total of 114 MDR strains were detected between 2016 and 2021, with a detection rate of 8.5% (114/1338). Among them, the MDR rate of *S. typhimurium* was the lowest at 6.9% (73/1053), and it was statistically significant compared to *S. enteritidis* (12/97, 12.4%) and other serotypes of *Salmonella* (*χ*^2 ^= 21.855, *P* < 0.01) (Table 5).

**Table 5:** Prevalence of multidrug-resistant (MDR) Salmonella isolates from 2016 to 2021.

Years	All serotypes of *Salmonella*		*S.* Typhimurium		*S.* Enteritidis		Other serotypes of *Salmonella*		χ^2^	*P* value
	MDR/Total	%	MDR/Total	%	MDR/Total	%	MDR/Total	%		
2016	1/94	1.1	1/77	1.3	0/13	0	0/4	0	21.86	<0.01
2017	9/131	6.9	3/101	3	2/11	18.2	3/19	15.8		
2018	69/234	29.5	44/197	22.3	6/13	46.2	18/27	66.7		
2019	7/228	3.1	4/187	2.1	1/15	6.7	2/25	8		
2020	5/335	1.5	4/256	1.6	0/20	0	1/57	1.8		
2021	23/316	7.3	17/235	7.2	1/25	4	5/56	8.9		
Total	114/1338	8.5	73/1053	6.9	12/97	12.4	29/188	15.4		

## Discussion

*Salmonella* is a zoonotic intestinal pathogen with many serotypes, mainly causing food-borne infections which lead to corresponding clinical symptoms (15). *Salmonella* infections are primarily divided into typhoidal and non-typhoidal (16). The former manifests as bloodstream infections, which are more common in adults; While the latter usually manifests as intestinal infections, which cause symptoms such as diarrhea, fever and abdominal pain and are more common in children (17, 18). This study demonstrated that the main specimen type of children with *Salmonella* infection was feces (92.75%), and fecal *Salmonella* isolation rate was increasing year by year, mainly in infants and toddlers under 3 years old (92.97%), consistent with domestic reports (19). This may be related to the fact that children are more susceptible than adults due to their lower immunity and weak gastrointestinal resistance (20). On the other hand, infant milk products have been reported as related to *Salmonella* outbreaks worldwide (21). This study also showed that *Salmonella* infections in children tend to have a high incidence in summer and autumn, which was consistent with domestic reports (22). However, the winter *Salmonella* infection rate in Guangzhou is still relatively high compared with that in northern China. This may be related to the geographical location of Guangzhou, which has a south subtropical maritime monsoon climate, with an average annual temperature of 21.5–22.2°C and abundant rainwater resources. Hot and humid weather promotes the growth of *Salmonella* and insufficient heat treatment of food can increase the risk of *Salmonella* infection.

In this study, we conducted a study of the clinical manifestations of *Salmonella* infection in children. 1,338 children with intestinal *Salmonella* infection had fever (62.18%) and diarrhea (60.16%) as prominent symptoms, but some children did not present with gastrointestinal symptoms. Previous reports of domestic *Salmonella* invasive infections mainly concentrated on typhoid or paratyphoid bloodstream infections (23, 24). In recent years, improvements in living standards and sanitation facilities have maintained *Salmonella* typhoid and paratyphoid infections at a low level. The increase in detection rate of invasive non-typhoid *Salmonella* (iNTS) has gradually attracted attention. The main diagnosis of invasive *Salmonella* infection is based on systemic multi-system clinical manifestations, symptoms of infection and poisoning, and *Salmonella* cultured in sterile body fluids (25). Extraintestinal infections caused by iNTS have also been reported, and the clinical features are similar to those of typhoid fever. It usually does not cause diarrhea, but causes bloodstream infections (such as bacteremia) or secondary systemic focal infections (such as meningitis), and failure to treat can often lead to death. The main reported serotypes are *typhimurium*, *enteritidis*, *dublin* and *swine* cholera. *S*. *typhimurium* ST313 is the main epidemic type of iNTS internationally, especially in Africa (26, 27). Specific susceptible populations of iNTS include HIV patients, immunodeficiency, hematological malignancies, young children, and malnourished or anemic adolescents, with a fatality rate as high as 20% (28). Unlike common NTS infections, patients rarely displayed gastroenteritis symptoms, but all had fever symptoms. Combined with the gradual increase in detection rate of *Salmonella* in recent years, the trend of increasing iNTS in Guangzhou cannot be ruled out. In addition, this study showed that there some children experienced diarrhea with clinical manifestations similar to viral diarrhea, no symptoms of high fever and diarrhea, routine stool microscopy, a significant increase in the number of stools, accompanied by obvious electrolyte metabolism disorders, and fecal culture detects *Salmonella* positive. Therefore, the necessity of early fecal culture detection in the diagnosis and treatment of children with diarrhea should be emphasized to avoid misdiagnosis.

The serotypes of 1,338 *Salmonella* strains in this study covered groups A to E, mainly group B (1110, 82.88%) and group D (124, 9.27%), of which *S*. *typhimurium* had the highest detection rate (1053, 78.69%), followed by *S*. *enteritidis* (7.17%) and *S*. *neurangium* (2.17%). The detection rate of *S*. *typhimurium* was higher than that reported by the CHINET Bacterial Resistance Surveillance Network (27.4%), and also higher than that of Tongji Hospital affiliated to Tongji Medical College of Huazhong University of Science and Technology (51.6%) and the Affiliated Children's Hospital to Zhengzhou University (36.5%) (29, 30). In addition, the serotypes detected in this study were different to serotypes collected in Wuhan, Beijing, Zhengzhou and other locations, indicative of regional differences in *Salmonella* serotypes. A total of 48 serotypes were detected in this study and the distribution was diversified, with some rare serotypes. Different *Salmonella* serotypes exhibit differences in antibiotic resistance. AMP resistance was mainly manifested in *Salmonella typhimurium*, *enteritidis*, *indiana* and *delby* as AMP was the earliest and most widely used antibiotic in livestock in China (31). The diversification of serotype distribution will lead to broadening of the MDR phenotype and antimicrobial resistance spectrum, and the trend of pan-drug resistance will become more evident. In previous studies, MDR strains mainly existed in *S*. *typhimurium*, but this study demonstrated that *S*. *typhimurium* had the lowest MDR rate among MDR *Salmonella*. Therefore, it is necessary to continuously and regularly monitor *Salmonella* serotypes in children to detect changes in the main types as early as possible.

Due to the excessive use of antibiotics in animal husbandry and clinical practice, *Salmonella* resistance is increasing, especially to AMP and 3rd generation cephalosporins (32). Generally, *Salmonella* has been resistant to previously used first-line antimicrobial drugs such as AMP, CHL, sulfamethoxazole, and amoxicillin. In this study, *Salmonella* presented the highest resistance rate against AMP of 84.5%, which was greater than other domestic scholars and regions to AMP. The resistance rate to 3rd generation cephalosporins (CRO, CTX, CAZ) was about 40.0%. *Salmonella* mainly produces ESBL, extended-spectrum *β*-lactamase and AmpC enzymes to generate resistance to cephalosporin antimicrobial drugs. Synthesis of *Salmonella* ESBL will indirectly reduce its sensitivity to quinolones, leading to the generation of multi-drug resistant strains resistant to cephalosporins and quinolones (33). Although the antimicrobial resistance rate of CIP in this study was low (6.7%), due to the side effects of central nervous and joint toxicity, it is no longer a suitable drug for children with *Salmonella* infection. CLSI 2020 recommends that only AMP, quinolones and trimethoprim-sulfamethoxazole are required for regular reports for fecal-isolated *Salmonella*. According to the antimicrobial sensitivity results of this study and the poor therapeutic effect of AMP in clinical use for *S*. *Typhimurium*, AMP is unsuitable for clinical promotion and application. At present, *Salmonella* still maintains high sensitivity to enzyme inhibitor compound preparations, 4th generation cephalosporins and IPM. When choosing antimicrobial treatment, drugs with antibacterial activity against *Salmonella* should be selected. Antimicrobial spectrum does not include gastrointestinal infections and drugs with unknown antimicrobial activity against *Salmonella* may lead to drug resistance. In order to decrease antibiotic consumption and AMR, the majority of gastroenteritis caused by *Salmonella* should not be treated with antibiotics (23, 34). In general, the current resistance rate of 3rd generation cephalosporins and quinolone antibiotics is still low, but there is an upward trend. Antimicrobial susceptibility studies to azithromycin are lacking.

There have been disagreements regarding the selection, use, and timing of antibiotics for *Salmonella* infection in children (35). At present, most of the 3rd generation cephalosporins are the first choice for clinical use, with azithromycin as an alternative. Quinolone antibiotics are generally not recommended for children, but can be considered for severe infections when no other antibiotics can be substituted. For MDR *Salmonella*, carbapenem antibiotics are suggested. The use of multiple antibiotics in combination is not recommended, and specific antibiotic selection should be based on the local epidemiology and antimicrobial susceptibility results. recommended course of treatment for intestinal infections is within 1 week, while the course of treatment for extra-intestinal infections needs to be extended. It is worth noting that there are few studies with large samples on the comparison of treatment effects between azithromycin, 3rd generation cephalosporins and quinolone antibiotics in children's NTS, and more extensive and in-depth research is required.

## Conclusion

In summary, under the current pressure of antibiotic selection, the typing and continuous monitoring of antimicrobial resistance of intestinal *Salmonella* is important research work to provide clear epidemiological evidence for clinical diagnosis, treatment, and prevention of infection.

## Data Availability

The original contributions presented in the study are included in the article/Supplementary Material, further inquiries can be directed to the corresponding author/s.
